# The Interaction between Coagulation Factor 2 Receptor and Interleukin 6 Haplotypes Increases the Risk of Myocardial Infarction in Men

**DOI:** 10.1371/journal.pone.0011300

**Published:** 2010-06-24

**Authors:** Bruna Gigante, Anna M. Bennet, Karin Leander, Max Vikström, Ulf de Faire

**Affiliations:** 1 Division of Cardiovascular Epidemiology, Institute of Environmental Medicine, Stockholm, Sweden; 2 Department of Medical Epidemiology and Biostatistics, Karolinska Institutet, Stockholm, Sweden; 3 Department of Cardiology, Karolinska University Hospital, Stockholm, Sweden; Universität Würzburg, Germany

## Abstract

The aim of the study was to investigate if the interaction between the coagulation factor 2 receptor (F2R) and the interleukin 6 (IL6) haplotypes modulates the risk of myocardial infarction (MI) in the Stockholm Heart Epidemiology Program (SHEEP). Seven SNPs at the F2R locus and three SNPs at the IL6 locus were genotyped. Haplotypes and haplotype pairs (IL6*F2R) were generated. A logistic regression analysis was performed to analyze the association of the haplotypes and haplotype pairs with the MI risk. Presence of an interaction between the two haplotypes in each haplotype pair was calculated using two different methods: the statistical, on a multiplicative scale, which includes the cross product of the two factors into the logistic regression model; the biological, on an additive scale, which evaluates the relative risk associated with the joint presence of both factors. The ratio between the observed and the predicted effect of the joint exposure, the synergy index (S), indicates the presence of a synergy (S>1) or of an antagonism (S<1). None of the haplotypes within the two loci was associated with the risk of MI. Out of 22 different haplotype pairs, the haplotype pair 17 GGG*ADGTCCT was associated with an increased risk of MI with an OR (95%CI) of 1.58 (1.05–2.41) (p = 0.02) in the crude and an OR of 1.72 (1.11–2.67) (p = 0.01) in the adjusted analysis. We observed the presence of an interaction on a multiplicative scale with an OR (95%CI) of 2.24 (1.27–3.95) (p = 0.005) and a slight interactive effect between the two haplotypes on an additive scale with an OR (95%CI) of 1.56 (1.02–2.37) (p = 0.03) and S of 1.66 (0.89–31). In conclusion, our results support the hypothesis that the interaction between these two functionally related genes may influence the risk of MI and suggest new mechanisms involved in the genetic susceptibility to MI.

## Introduction

The study of interactions between genes coding for proteins involved in common metabolic pathways might shed light on the complex mechanisms underlying the pathogenesis of complex diseases.

A large body of evidence indicates that the interplay between thrombosis and inflammation plays a crucial role in the progression of atherosclerotic plaque growth and may drive the plaque towards rupture or stabilization [1]. The thrombin receptor, the coagulation factor 2 receptor (F2R), is a critical modulator of inflammation and thrombosis crosstalk in the vessel wall [2]. Studies performed in vitro have demonstrated that in endothelial cells thrombin stimulates a plethora of pro-inflammatory mediators thus exerting a pro-atherogenic effect [3], [4], [5]. In particular, F2R stimulates the synthesis of interleukin 6 (IL6) a cytokine exerting strong pro-atherogenic effects that mediate the transition of the inflammatory process in the vascular wall from an acute to a chronic phase [6]. Increased levels of serum IL6 represent an established risk factor for coronary heart disease [7], [8].

Recently, we have shown that a promoter (−1738 G/A) and an intronic (2860 G/A) F2R genetic variant and their related haplotypes are associated with higher IL6 serum levels and modulate the risk of myocardial infarction (MI) in male patients through the interaction with IL6 serum levels [9].

The aim of this study was to analyze the presence and the potential role of an interaction between F2R and IL6 haplotypes on the risk of MI in a large case control population recruited in the Stockholm area, the Stockholm Heart Epidemiology Program (SHEEP).

## Results

Pairwise linkage disequilibrium (LD) values between F2R SNPs in the SHEEP have been already reported [9]; LD values between SNPs within the IL6 gene are as follows: rs1800795–rs1800796 and rs1800795–rs2069840 r^2^ = 0.051 [7] and 0.03, respectively; rs1800796–rs2069840 r^2^ = 0.36).

**Figure 1:**
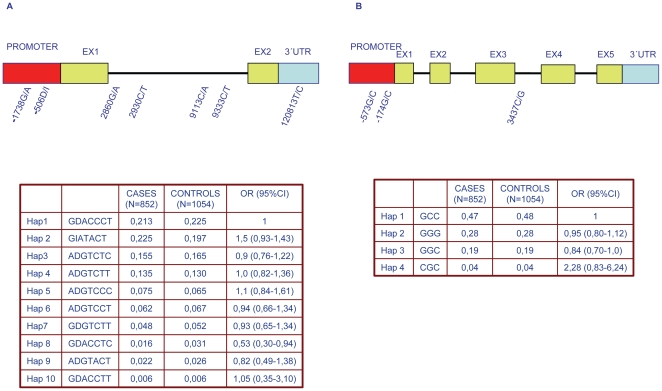
F2R and IL6 genes: tag SNPs and haplotype frequencies. Top panel. Schematic representation of F2R (Panel A) and IL6 (Panel B) tagSNPs mapping position. Ex: exon, 3′UTR: 3′untranslated region. Bottom panel. Table illustrating F2R (Panel A) and IL6 (Panel B) haplotype frequency in SHEEP male cases and controls. The table reported in panel A has been modified from Gigante B. et al, *Thromb Haemost*. 2009;101(5):943–953 (permission to reprint obtained from the publisher).

None of the F2R and IL6 haplotypes were associated with the risk of MI in men in the SHEEP study (Figure 1A and 1 B, bottom panel).

The twenty-two most common haplotype pairs (IL6*F2R) in men are listed in Table 1. Haplotype pair 17 (GGG*ADGTCCT) was more frequent in cases (p = 0.04), while haplotype pairs 14,15 and 16 were observed more often in controls although the difference fell short of significance.

**Table 1:** Absolute frequency of IL6*F2R haplotype pairs in male cases and controls.

*Pair*	*IL6 Hap*	*F2R Hap*	*Cases (%)*	*Controls (%)*
**1**	**GCC**	**GDACCCT**	227 (10.4)	289 (10.7)
**2**	**GCC**	**GIATACT**	236 (10.8)	274 (10.2)
**3**	**GCC**	**ADGTCTC**	201 (9.2)	233 (8.7)
**4**	**GGG**	**GDACCCT**	151 (6.9)	204 (7.6)
**5**	**GGG**	**GIATACT**	153 (7)	183 (6.8)
**6**	**GCC**	**ADGTCTT**	131 (6)	153 (5.7)
**7**	**GGG**	**ADGTCTC**	132 (6)	153 (5.7)
**8**	**GGC**	**GDACCCT**	113 (5.1)	144 (5.4)
**9**	**GGC**	**GIATACT**	113 (5.2)	124 (4.6)
**10**	**GGG**	**ADGTCTT**	93 (4.2)	118 (4.3)
**11**	**GGC**	**ADGTCTC**	88 (4)	115 (4.3)
**12**	**GCC**	**ADGTCCT**	81 (3.7)	99 (3.7)
**13**	**GCC**	**ADGTCCC**	79 (3.6)	84 (3.1)
**14**	**GCC**	**GDGTCTT**	58 (2.6)	91 (3.3)
**15**	**GGC**	**ADGTCTT**	52 (2.3)	83 (3)
**16**	**GGG**	**GDGTCTT**	33 (1.5)	61 (2.2)
**17**	**GGG**	**ADGTCCT**	62 (2.9)	55 (2)
**18**	**GGG**	**ADGTCCC**	54 (2.4)	56 (2)
**19**	**GGC**	**ADGTCCT**	31 (1.4)	53 (1.9)
**20**	**GCC**	**ADGTACT**	25 (1.1)	42 (1.5)
**21**	**GGC**	**ADGTCCC**	37 (1.7)	39 (1.4)
**22**	**GCC**	**GDACCTC**	29 (1.3)	31 (1.1)

We performed a logistic regression analysis to test if these four haplotype pairs were associated with the risk of MI. As shown in Table 2, the haplotype pair 14 was associated with a reduction in the MI risk at the adjusted analysis while the presence of the haplotype pair 17 was associated with an increased risk of MI with an OR of 1.58 (1.05–2.41) at the crude and an OR of 1.72 (1.11–2.67) at the adjusted analysis.

**Table 2:** Estimated effect of haplotype pairs 14, 15, 16 and 17 on the risk of MI.

	*Crude* *		*Adjusted* **	
	OR (95%CI)	P	OR (95%CI)	P
Pair 14 (GCC*GDGTCTT)	0.77 (0.55–1.09)	0.14	0.69 (0.48–0.99)	0.04
Pair 15 (GGC*ADGTCTT)	0.79 (0.55–1.14)	0.20	0.80 (0.55–1.17)	0.25
Pair 16 (GGG*GDGTCTT)	0.66 (0.42–1.02)	0.06	0.62 (0.37–1.04)	0.07
Pair 17 (GGG*ADGTCCT)	1.58 (1.05–2.41)	0.02	1.72 (1.11–2.67)	0.01

*Adjusted for age and hospital catchment area.

**Adjusted for age, hospital catchment area, hypertension, diabetes, smoking, body mass index >30 and hypercholesterolaemia.

To investigate the mechanisms underlying this observation we tested the hypothesis of an interaction between IL6 and F2R haplotypes in the haplotype pair 17 (Table 3) and 14. Under the assumption of a biological interaction, joint presence of the IL6 and F2R haplotypes was associated with an OR (95%CI) of 1.56 (1.02–2.37). The calculation of the S (95%CI) gave a value of 1.66 (0.89–31). When the interaction between these two haplotypes was tested on a multiplicative scale we observed that the cross product of these two haplotypes was associated with an OR of 2.24 (1.27–3.95) (p = 0.004). No significant interaction between the two haplotypes at haplotype pair 14 was observed either under a biological [OR 0.77 (0.50–1.17), p = 0.22] or a statistical [OR 0.66 (0.35–1.22), p = 0.18] model.

**Table 3:** Interaction between the IL6 Hap2 and F2R Hap6 on a multiplicative and on an additive scale.

	*N*	*OR (95%CI)*	P
IL6HapGGG	633	0.91 (0.74–1.12)	0.38
F2R HapADGTCCT	123	0.75 (0.51–1.10)	0.15
**Multiplicative**			
IL6XF2R (Pair 17)	98	2.24 (1.27–3.95)	0.005
**Additive**			
IL6+F2R (Pair 17)	98	1.56 (1.02–2.37)	0.03

All individuals homozygotes for IL6 HapGGG (n = 18) or for F2R HapADGTCCT (n = 1) have been excluded from the interaction analysis.

## Discussion

Haplotypes may be considered as “super-alleles” where the different genetic variants encoded act in synergy and contribute to determine the biological function of a protein, through changes in its primary, secondary or tertiary structure [10]. In this perspective haplotypes may have a greater power to detect the association of candidate genes with a complex phenotype by either capturing the effect of un-genotyped causal SNPs or, in the presence of several SNPs each one exerting a small effect hardly detected when the single variants are analyzed separately, by identifying new candidate genes and/or genetic variants [11], [12].

Studies performed so far in vitro on the adrenergic receptors have suggested that different haplotypes might regulate selective biological pathways and physiological responses to different stimuli [12]. More recently we have also shown that selective F2R haplotypes are associated with peculiar phenotypes [9], [13]. In the present study we sought to investigate if IL6 and F2R haplotype pairs were associated with the risk of MI. This hypothesis was driven by our former study showing that two F2R SNPs modulate IL6 serum levels and interact with IL6 serum levels in determining the risk of MI [9], suggesting the presence of an interaction between IL6 and F2R genes. The modifying effect on the risk of a disease exerted by the concomitant presence of two haplotypes, encoding the same gene or two genes involved in the same metabolic pathway has been already reported, among others, in a study of the Islandic population where the selective haplotype combinations within the gene encoding phosphodiesterase 4D were found to confer a differential risk of stroke [14] and in the German MI family study where haplotypes within the ghrelin gene were associated with a reduced risk of MI only in the presence of selective haplotypes encoding the ghrelin receptor, thus suggesting the presence of an epistatic interaction between these two genes [15]. Our results are in line with these observations indicating that the analysis of the association of haplotypes coding different genes may disclose the presence of a genetic effect that it is not readily observed when analyzing each gene separately. Given the complexity of the mechanisms through which genes affect the occurrence of complex traits, it has been hypothesized that the presence of one or more haplotypes in different genes involved in the same metabolic pathways of importance for the progression of a disease may influence the genetic load of an individual thus increasing or decreasing the individual susceptibility to suffer from complex disorders [16].

Different approaches have been undertaken to analyze the observed interaction effect [15], [16] between haplotypes or to investigate the presence of potential haplotypes interactions to partly explain the genetic variance in complex traits [17]. We tested our hypothesis under two different interaction models, a biological model that measures on an additive scale the risk difference between different exposures, and a statistical model on a multiplicative scale that considers the difference in the relative risk of a disease in the presence of both factors [18]. In the biological model we observed a borderline additive interaction effect on the risk of MI in individuals carrying the haplotype pair 17 as shown by the wide confidence interval relative to the S index; in the statistical interaction model we did observe a significant interaction between the two haplotypes. The results obtained by these two models are not readily comparable since they evaluate two different aspects of the interaction between two factors [19]. Presence of a multiplicative and absence of an additive interaction among two correlated genes has been already described [20] and probably reflects the complexity of the interaction between two genes in multifactorial diseases. The presence of an interaction on a multiplicative scale may reflect the role of other genes and/or environmental factors that participate in the same metabolic pathway and are therefore indirectly involved in determining the observed interactive effect.

Several limitations of the present study need to be acknowledged. The number of individuals in the rarest haplotypes is rather small and this may result in the loss of power in the interaction analysis; the replication of haplotype association studies is limited by the diversity of the haplotype structure of the same gene among different populations [9], [21] and finally in vitro or ex vivo studies are needed to obtain the biological proof of different effects related to different haplotypes within the same gene.

In conclusion, our results support the hypothesis that a genetic interaction between the thrombin receptor and the interleukin 6 gene plays a role in the occurrence of myocardial infarction. In a broader perspective, our results suggest new mechanisms to be further explored in the analysis of the association of genes with complex traits.

## Methods

### Study Population

SHEEP was designed as a population based case-control study to dissect both genetic and environmental factors underlying the occurrence of MI and to compare the effects of the different risk factors in men and women [22].

Briefly, SHEEP comprises Swedish citizens living in the Stockholm County who were 45–70 years of age and free of previous MI. Cases were identified during the period 1992 to 1994 and only patients who survived at least 28 days after the MI event were included in the present study (n = 1213). One control per case was randomly selected from the Stockholm County population registry after stratification for age (with a 5-years interval), sex and hospital catchment area. In addition other 5 controls were selected at the same time to replace eventual non-responders. When the initial control replied late, both the initial and the already enrolled substitute control have been included in the study. This resulted in the inclusion of more controls (n = 1561) than cases. A more detailed description of the SHEEP population has been reported elsewhere [9], [22] The study was approved by the Ethics Committee at the Karolinska Institutet (Stockholm, Sweden).

### Ethics

The Ethical Committee at Karolinska Institutet approved the SHEEP study design in 1991 (Protocol Number 1991, 91:259). All the study participants gave their informed oral consent to be enrolled in the study, since at the time the study was initiated (1992) no forms for the written consent were available or in current use. The Ethical Committee at Karolinska Institutet has then approved molecular genetic analyses to be performed on the SHEEP material in 2001 (Protocol Number 2001, 01-097).

### Tag Single Nucleotide Polymorphisms (tSNPs) selection and genotyping and haplotypes generation

The following 7 tSNPs mapping within F2R gene were selected through HapMap *(* www.hapmap.org *)* and genotyped as previously described [9], [13]: −1738 G/A (rs2227744), −506D/I (rs11267092) in the promoter, 2860 G/A (rs27135), 2930 C/T (rs27593) and 9113 C/A (rs2227774), 9333C/T (rs37250) in the intron, and 1801719T/C (rs1801719 ) in the 3′untranslated region (Figure 1A, top panel) . Three tSNPs were selected through HapMap for the IL6 gene −573 G/C (rs1800795) and −174G/C (rs1800796) in the promoter were genotyped as previously described [7], and 3437C/G (rs2069840) in the 3^rd^ intron (Figure 1B, top panel) was genotyped by Taqman with a custom made assay purchased from Applied Biosystems (ABI).

Haplotypes were generated from the original genotype data using the software PHASE v2 [23].

### Statistical Analysis

Based on our former results showing that the effects of F2R on MI risk is absent among women [9], the present study was restricted to an all male population. For each SNP, concordance to the Hardy-Weinberg equilibrium was tested by 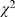 test with 1DF.

The pairwise LD values between the SNPs were assessed by THESIAS [24].

We tested the association of the F2R and the IL6 haplotypes, generated from the original genotype data, with myocardial infarction by THESIAS [24]. The algorithm used in THESIAS takes the most commonly observed haplotype in the population under investigation as the reference category and expresses the risk associated with the presence of each one of the other haplotypes as odds ratios (ORs) with 95% confidence intervals (CI) [(ORs (95% CI)]. For the purpose of our analysis crude OR (95%CI) were adjusted for the study design variables age and hospital catchment area.

We have then calculated the absolute frequency of each haplotype pair (IL6* F2R) taking into account all the possible haplotype pairs for each individual. The following inclusion/exclusion criteria were followed: haplotypes with more than 3 dropouts for F2R and IL6 genotypes were excluded, only individuals with complete haplotype information for both genes were included and only haplotype pairs with a frequency ≥1% have been included in the analysis. The analysis was then performed on 1728 individuals. To correct for the analysis of multiple SNPs within a gene we have used a gene-wide significance level (P) that takes into account the overall genetic variation in the genes under investigation [25]. The actual p value is corrected according to the ratio (r) SNPs tested/all SNPs validated within a gene applying the formula P = 1−(1−0.05)^r^. According to the most recent version of HapMap (Feb09) 17 SNPs have been validated within the F2R gene and 10 in the IL6 gene. Of these 5/17 and 5/10 were found to be in high LD (r^2^>0.95) and therefore not included in the present analysis giving a threshold p value of 0.03 for both genes.

The difference in the frequency of IL6 and F2R haplotypes pair (IL6* F2R) between cases and controls has been tested by χ^2^ test with 1DF. The association of the each haplotype pair with MI has been performed through a logistic regression analysis and the results expressed as odds ratio (OR) and 95% confidence interval (95%CI). The crude OR (95%CI) were adjusted for the study design variables (indicated as crude) and further adjusted for hypertension (defined as blood pressure values>140/90 mmHg or use of an anti-hypertensive medication), diabetes (blood glucose levels>6.7 mmol/L or diabetes controlled with diet, oral drug treatment or insulin), smoking (current smoking within the last two years), body mass index >30 and hypercholesterolemia (serum level of total cholesterol ≥6.46 mmol/L or treatment with lipid lowering drugs) in the multivariate analysis (indicated as adjusted).

We have then explored the presence of an interaction effect between the haplotypes within haplotype pairs associated with MI using two different methods: a biological, on an additive scale, and a statistical, on a multiplicative scale. To estimate the presence of a biological interaction between the two haplotypes in a specific haplotype pair, we have estimated by logistic regression analysis the risk, expressed as OR (95%CI), associated with the presence of one haplotype in the absence of the other one (i.e. IL6 GGG but not F2R ADGTCCT [AO] and F2R ADGTCCT but not IL6 GGG [OB]) and the risk associated with the presence of both haplotypes (i.e. GGG+ADGTCCT [AB]) taking as reference group all the individuals not exposed to either or both haplotypes [OO]. The presence of synergism or antagonism between two factors is expressed by the synergy index (S) 95% CI. The presence of a S value higher than 1 indicates the presence of a synergism between the two haplotypes, the presence of a S value lower than 1 indicates the presence of an antagonism between the two haplotypes [26]. The formula to calculate S has been reported by Rothman [27] and derives from the ratio between the risk (R) observed in the presence of both haplotypes minus the risk observed in the reference group and the risk predicted by the sum of the risk observed in individuals exposed to only one factor minus twice the risk in the reference group (i.e. RAB-ROO/RAO+ROB-2XROO).

To estimate the presence of a statistical interaction the cross product of the two factors was included as a variable in the logistic regression model.

Calculations were carried out using SAS (vers 9.1, SAS Institute Inc. Cary, NC).
